# Enhanced efficiency of dye-sensitized solar cells doped with green phosphors LaPO_4_:Ce, Tb or (Mg, Zn)Al_11_O_19_:Eu

**DOI:** 10.1186/1556-276X-8-219

**Published:** 2013-05-08

**Authors:** Chang Kook Hong, Hyun-Seok Ko, Eun-Mi Han, Je-Jung Yun, Kyung-Hee Park

**Affiliations:** 1 School of Applied Chemical Engineering, Chonnam National University, Gwangju, 500-757, Republic of Korea; 2 Department of Advanced Chemicals & Engineering, Chonnam National University, Gwangju, 500-757, Republic of Korea; 3 Jeonnam Nano Bio Research Center, Jangseong-gun, Jeollanam-do, 515-893, Republic of Korea; 4 The Research Institute for Catalysis, Chonnam National University, Gwangju, 500-757, Republic of Korea

**Keywords:** Green phosphor, Dye-sensitized solar cell, Photoluminescence, Conversion efficiency

## Abstract

We have successfully introduced green phosphors LaPO_4_:Ce, Tb (G4) or (Mg, Zn)Al_11_O_19_:Eu (G2) into TiO_2_ photoelectrode of dye-sensitized solar cells. The conversion efficiency of the G4-doped device was enhanced by 30% compared with the pristine TiO_2_ photoelectrode. The green phosphor doped at 5-wt.% ratio contributed to the reduction of resistances of the surface and interface of the photoelectrode and to the great enhancement of the absorption spectrum in UV-visible and near-infrared regions. The internal resistances and absorbance of the photoelectrode directly affect the power conversion efficiency. Green phosphor plays an important role towards the realization of high-efficiency dye-sensitized solar cells.

## Background

The uses of different types of nanostructured materials in dye-sensitized solar cells (DSSC) have attracted worldwide attention as a low-cost alternative to traditional photovoltaic device [1-5]. This is because nanostructures of materials enhance the surface area to allow a higher amount of dye molecules to be adsorbed, and the nature of electron transport in oxide nanoparticle films is fairly well understood. The scientific community is still struggling to find optimum nanostructures and materials for the best solution to overcome issues associated with stability, efficiency, and cost-effective mass production [6,7].

Normally, in DSSCs, photons interact with dye molecules to create excitons. These excitons come into contact with nanoparticles/nanostructures at the surface of the photoelectrode and are rapidly split into electrons and holes. Electrons are injected into the photoelectrode, and holes leave the opposite side of the device by means of redox species (traditionally the I^−^/I_3_^−^ couple) in the liquid or solid-state electrolyte used in DSSCs to ensure efficient electron transfer to the redox couple [8-11]. It is important to apply different materials and structures to enhance light photon interaction with dye molecules to achieve a higher proportion of excitons.

Recent research shows that the implementation of a UV-absorbing luminescent wavelength converter (which is emitted at longer wavelengths) not only remarkably improves the photochemical stability of the DSSCs but also enhances the efficiency of the cell [3,11-14].

LaPO_4_:Ce, Tb (G4) and (Mg, Zn)Al_11_O_19_:Eu (G2) have been widely used in tricolor phosphor lamps and PDP displays as highly effective green phosphor additives [15-18]. YVO_4_:Bi^3+^, Ln^3+^ (Ln = Dy, Er, Ho, Eu, and Sm) phosphors are proposed to be promising UV-absorbing spectral converters for DSSCs as they possess broad absorption band in the whole UV region of 250 to 400 nm and could emit intense visible lights. When excited by ultraviolet light, G4 emits 550 nm of light in the green region. Considering this point, the doping of green phosphors LaPO_4_:Ce, Tb or (Mg, Zn)Al_11_O_19_:Eu into TiO_2_ photoelectrodes could lead to higher efficiency in dye-sensitized solar cells. Field emission-scanning electron microscopy (FE-SEM) was used to determine the morphology of this hybrid photoelectrode. The absorption and luminescence properties of dye and green phosphor ceramics were investigated using UV spectrophotometry and photoluminescence spectrometry. Electrochemical measurements were used to optimize the weight percentage of fluorescent materials doped in TiO_2_ photoelectrode, which had higher conversion efficiency (*η*), fill factor (FF), open-circuit voltage (*V*_oc_), and short-circuit current density (*J*_sc_) as a result.

## Methods

### Materials

Anhydrous LiI, I_2_, poly(ethylene glycol) (mw = 20,000), nitric acid, and 4-tertiary butyl pyridine were obtained from Sigma-Aldrich (St. Louis, MO, USA), and TiO_2_ powder (P25) was obtained from Nippon Aerosil (EVONIK Industries AG, Hanau-Wolfgang, Germany) and used as received. Ethanol was purchased from Daejung Chemicals & Metals Co. (Shiheung, Republic of Korea), and water molecules were removed by placing molecular sieves (3 Å) in the solvent. Commercially sourced bis(isothiocyanato)bis(2,2′-bipyridyl-4,4′-dicarboxylato)-ruthenium(II)-bis-tetrabutyl ammonium (N719 dye) and 1,2-dimethyl-3-propylimidazolium iodide were obtained from Solaronix SA (Aubonne, Switzerland). Green phosphors LaPO_4_:Ce, Tb and (Mg, Zn)Al_11_O_19_:Eu were obtained from Nichia Corporation (Tokushima, Japan). The electrolyte solution consisted of 0.3 M 1,2-dimethyl-3-propylimidazolium iodide, 0.5 M LiI, 0.05 M I_2_, and 0.5 M 4-*tert*-butylpyridine in 3-methoxypropionitile.

### Fabrication of DSSC

TiO_2_ powder was thoroughly dispersed for 10 h at 300 rpm using a ball mill (Planetary Mono Mill, FRITSCH, Oberstein, Germany), adding acetyl acetone, poly(ethylene glycol), and a Triton X-100 to obtain a viscous TiO_2_ paste. The doped green phosphors were added to the TiO_2_ paste and mixed in a ball mill for 2 h. The TiO_2_ and green phosphor-doped TiO_2_ pastes were coated onto fluorine-doped SnO_2_ conducting glass plates (FTO, 8 Ω cm^−2^, Pilkington, St. Helens, UK) using squeeze printing technique, followed by sintering at 450°C for 30 min. Approximately 8- to 10-μm-thick TiO_2_ film was deposited onto a 0.25-cm^2^ FTO glass substrate. Glass-FTO/TiO_2_ and phosphor-doped TiO_2_ electrodes were immersed overnight (*ca*. 24 h) in a 5 × 10^−4^ mol/L ethanol solution of Ru(dcbpy)_2_(NCS)_2_ (535-bis TBA, Solaronix), rinsed with anhydrous ethanol, and dried. A few drops of the liquid electrolyte were dispersed onto the surface, and a full cell assembly was constructed for electrochemical measurements. A Pt-coated FTO electrode was prepared as a counter electrode with an active area of 0.25 cm^2^. The Pt electrode was placed over the dye-adsorbed TiO_2_ thin film electrode, and the edges of the cell were sealed with 5-mm wide strips of 60-μm-thick sealing sheet (SX 1170–60, Solaronix). Sealing was accomplished by hot-pressing the two electrodes together at 110°C.

### Characterization of DSSC

The surface morphology of the film was observed by FE-SEM (S-4700, Hitachi High-Tech, Minato-ku, Tokyo, Japan). A 450-W xenon lamp was used as light source for generating a monochromatic beam. Calibration was performed using a silicon photodiode, which was calibrated using an NIST-calibrated photodiode G425 as a standard. UV-visible (vis) spectra of the TiO_2_ film and TiO_2_ electrode with green phosphor powder added were measured with a UV–vis spectrophotometer (8453, Agilent Technologies, Inc., Santa Clara, CA, USA). Photoluminescence spectra were recorded on Avantes BV (Apeldoorn, The Netherlands) spectrophotometer under the excitation of Nd:YAG laser beam (355 nm). Electrochemical impedance spectroscopies of the DSSCs were measured with an electrochemical workstation (CHI660A, CH Instruments Inc., TX, USA). The photovoltaic properties were investigated by measuring the current density-voltage (*J-V*) characteristics under irradiation of white light from a 450-W xenon lamp (Thermo Oriel Instruments, Irvine, CA, USA). Incident light intensity and active cell area were 100 mW cm^−2^ and 0.25 cm^2^, respectively.

## Results and discussion

**Figure 1:**
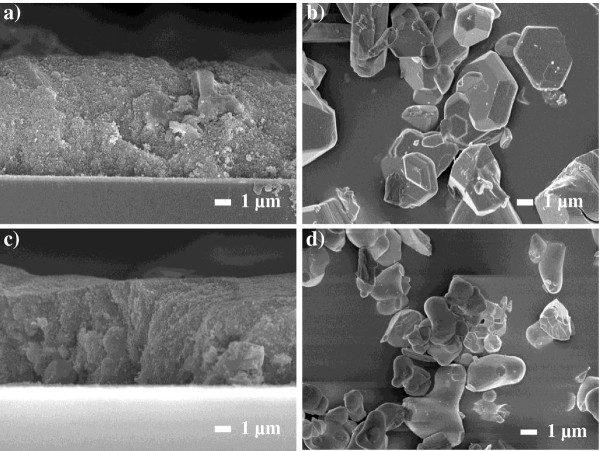
**Cross-sectional FE-SEM images of TiO**_**2 **_**electrode.** It is doped with 5 wt.% of G2 (**a**), G2 powder (**b**), TiO_2_ electrode doped with 5 wt.% of G4 (**c**), and G4 powder (**d**).

Figure 1 shows FE-SEM cross-sectional images of a TiO_2_ electrode doped with 5 wt.% of G2 (Figure 1a), G2 powder (Figure 1b), and a TiO_2_ electrode doped with 5 wt.% G4 (Figure 1c) and G4 powder (Figure 1d). The size of the two green phosphor powder particles varied from 3 to 7 μm without uniformity. These nonuniform micro-sized structures of the fluorescent powder could create porous and rough surface morphologies on the surface of and within the TiO_2_ photoelectrode. However, the maximum doping ratio was 5 wt.%. This type of structure has advantages for the adsorption of a higher percentage of dye molecules and also supports deeper penetration of the I^-^/I_3_^-^ redox couple into the TiO_2_ photoelectrode.

**Figure 2:**
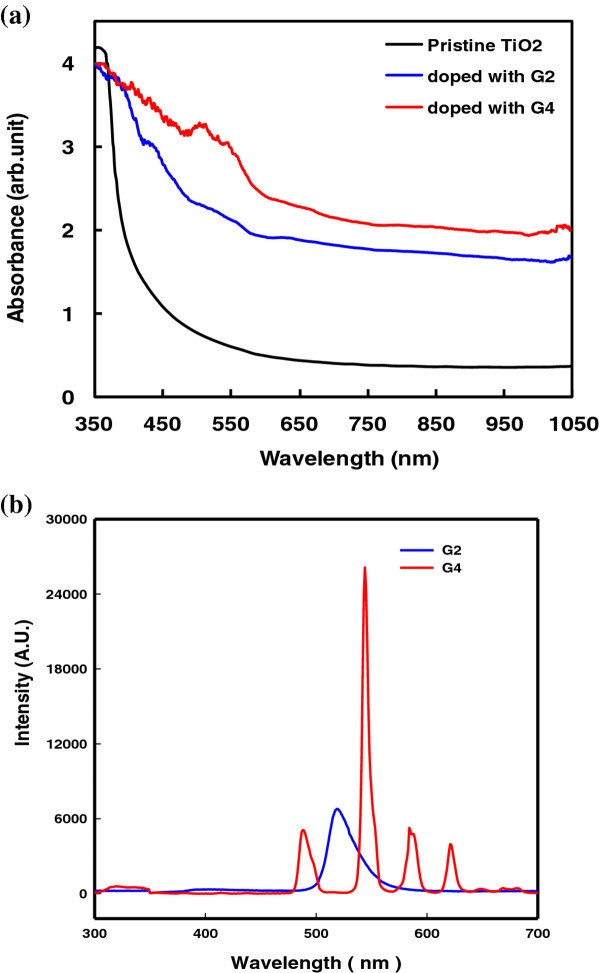
**Absorption of TiO**_**2 **_**electrode and emission spectra of G2 and G4.** (**a**) Absorption spectra of pristine TiO_2_ electrode. TiO_2_ electrode doped with 5 wt.% of G2, and TiO_2_ electrode doped with 5 wt.% of G4. (**b**) Emission spectra of G2 and G4.

Figure 2a shows the absorption spectra of a pristine TiO_2_ photoelectrode (black curve), a TiO_2_ photoelectrode doped with 5 wt.% G2 (blue curve), and a TiO_2_ photoelectrode doped with 5 wt.% G4 (red curve). The electrodes listed in the order of active absorption area are G4-doped photoelectrode > G2-doped photoelectrode > pristine TiO_2_ photoelectrode. The absorption spectra indicate that more photon energy could be harvested. The effective spectrum ranges from 375 to 900 nm. These spectra cover a UV-visible-IR region. The emission spectra of G2 and G4 are shown in Figure 2b, which was obtained by excitation at 254 nm with the emission line at 517 nm for G2 and excitation at 288 nm with the emission line at 544 nm for G4. To determine the optimal contents of the dopant, optoelectric and electrochemical technology were used. The optimal content of green phosphor was 5 wt.%.

**Figure 3:**
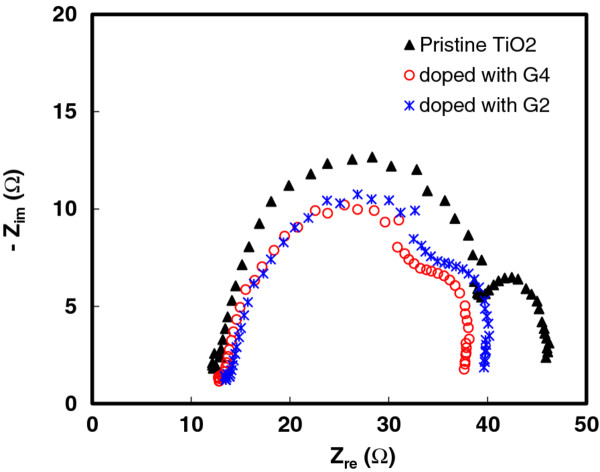
**Nyquist plot of the impedance characteristics between *Z***_**re **_**and *Z***_**im**_**.** It is with the angular frequency *ω* = 2π*f* of pristine TiO_2_ electrode and TiO_2_ electrode doped with 5 wt.% of G2 and TiO_2_ electrode doped with 5 wt.% of G4.

Figure 3 shows electrochemical impedance spectroscopy measurements for pristine, G2-doped, and G4-doped TiO_2_ photoelectrode. In these observations, the Nyquist plots of the impedance characteristics were obtained from the dependence of the real axis resistance (*Z*_re_) and imaginary axis resistance (*Z*_im_) along with the angular frequency. The diameter of the first semicircle at middle frequency illustrated in the spectra shows the charge-transfer resistance (*R*_ct_) between the TiO_2_ (or doped TiO_2_ with G2 and G4) and electrolyte. The bulk resistances (*R*_s_) of the pristine, G2-doped, and G4-doped TiO_2_ electrodes are 12.8, 13.7, and 13.4 Ω, respectively. The *R*_ct_ values of the pristine, G2-doped, and G4-doped TiO_2_ electrode devices are 26.3, 21.9, and 19.8 Ω, respectively. In the case of G4-doped TiO_2_ devices, smaller *R*_ct_ means a decrease in interfacial resistance and an increase of energy conversion efficiency. The results show a significant effect on the internal resistance of the solar cell and, consequently, can affect the fill factor and conversion efficiency.

**Figure 4:**
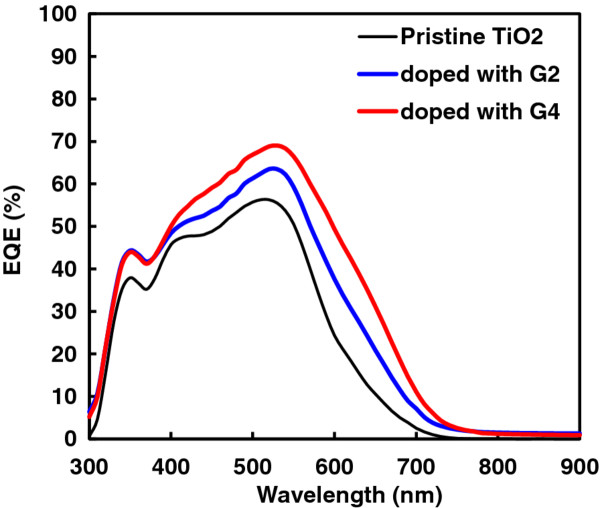
**IPCE spectra of pristine, doped with 5 wt.% G2, and 5 wt.% G4 TiO**_**2 **_**electrodes.**

The incident photon-to-current conversion efficiency (IPCE) spectra show the cell of a pristine TiO_2_ photoelectrode doped with 5 wt.% G2 and 5 wt.% G4. The pristine TiO_2_ photoanode exhibits a maximum IPCE value of 55% at 530 nm, while for the cell with TiO_2_ photoanode doped with G2 and G4, the peaks reach 65% and 70%, respectively, as shown in Figure 4. Moreover, an increase of IPCE value in the range of 550 to 650 nm for the cells with doped G2 and G4 photoanodes are observed due to the scattering effect of the G2 and G4 materials, which favor the improvement of *J*_sc_ for the cell [19]. The increase in *J*_sc_ with the amount of luminescent powder like G2 and G4 are due to the longer wavelength absorbed which transfers to visible light (550 to 650 nm). Also, larger particle sizes in G2 and G4 powders can extend the light transmission distance, improving incident light harvest and increasing the photocurrent [20].

The photoelectrochemical performance factors such as the FF and overall *η* were calculated by the following equations:

(1) $$\mathrm{FF}=\frac{J_{\max}\times V_{\max}}{J_{\mathrm{sc}}\times V_{\mathrm{oc}}},$$

(2) $$\begin{array}{l} \eta \left(\%\right)=\frac{p_{\mathrm{out}}}{p_{\mathrm{in}}}\times 100=\frac{J_{\max}\times V_{\max}}{P_{\mathrm{in}}}\times 100\\ \;=\frac{J_{\mathrm{sc}}\times V_{\mathrm{oc}}\times \mathrm{FF}}{P_{\mathrm{in}}}\times 100, \end{array}$$

where *J*_sc_ is the short-circuit current density (mA cm^−2^), *V*_oc_ is the open-circuit voltage (V), *P*_in_ is the incident light power, and *J*_max_ (mA cm^−2^) and *V*_max_ (V) are the current density and voltage in the *J-V* curve at the point of maximum power output, respectively.

**Figure 5:**
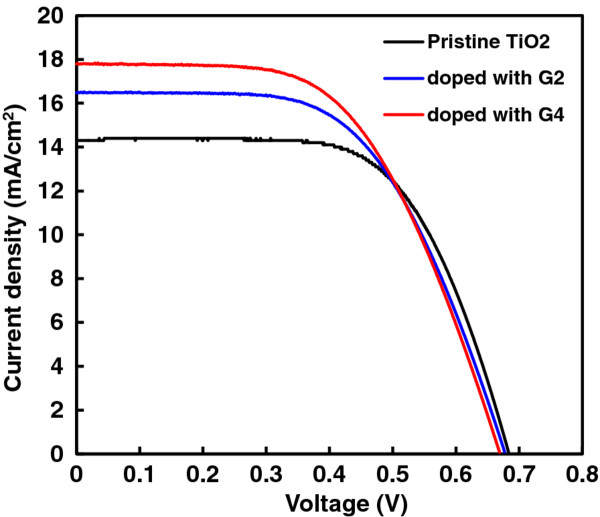
***J-V *curves of dye-sensitized solar cell.** It is based on pristine TiO_2_ electrode (**a**), TiO_2_ electrode doped with 5 wt.% G2, and TiO_2_ electrode doped with 5 wt.% G4.

Figure 5 shows *J*_sc_ versus *V*_oc_ characteristics of the DSSCs. The photoelectrochemical performance was measured by calculating *η*. The best conversion efficiency was 7.98% for the G4-doped device with a *J*_sc_ of 17.8 mA cm^−2^, a *V*_oc_ of 0.67 V, and an FF of 0.67. The pristine TiO_2_ and G2-doped device efficiencies were 6.15% and 7.16%, respectively. The open-circuit voltage changed slightly with the insertion of green phosphor, from 0.67 to 0.68 V, while the fill factor changed with the insertion from 0.63 to 0.67, and the short-circuit current changed from 14.3 to 17.8 mA cm^−2^. For pristine TiO_2_, *η* was 6.15%, which increased to 8.0% for 5 wt.% fluorescent powder added to TiO_2_ (Table 1). The effect of different weight percentage ratios of fluorescent powder added to the TiO_2_ was also investigated, and 5 wt.% was the optimum ratio. The DSSC with only TiO_2_ had lower *J*_sc_ and *V*_oc_ because it has a lower proportion of excitons. When the fluorescent powder was added, the number of photons increased and hence increased the probability of photon and dye molecule interactions. Our results suggest that the insertion of green phosphor provides optimal electron paths by reducing the surface and interface resistance, by changing the surface morphology of the electrode. Efficiency was increased by a factor of 2.

**Table 1:** **Photovoltaic properties of pristine TiO**_**2**_-**based DSSC and those doped with G2 and G4**

**Samples**	***V***_**oc**_	***J***_**sc**_	**FF**	***η***	***λ***_**ex**_	***λ***_**em**_
	**(V)**	**(mA cm**^**−2**^**)**		**(%)**	**(nm)**	**(nm)**
Pristine TiO_2_	0.68	14.30	0.63	6.15	-	-
Doped with G2	0.68	16.50	0.64	7.16	254	517
Doped with G4	0.67	17.80	0.67	7.98	288	544

Photovoltaic properties include open-circuit voltage (V), short-circuit current density (mA cm^−2^), fill factor, power conversion efficiency (%), excitation wavelength (nm), and emission wavelength (nm).

## Conclusions

In summary, we have successfully introduced a 5-wt.% ratio of green phosphors G4 or G2 into the TiO_2_ photoelectrodes of dye-sensitized solar cells. The enhanced percentage of conversion efficiencies of devices doped with G4 or G2 were 30% and 16% with the open-circuit voltages of 0.67 and 0.68 V and the short-circuit currents of 17.8 and 16.5 mA cm^−2^, respectively. The fill factors were 0.67 and 0.64, respectively. The 5-wt.% doping ratio of green phosphor contributed to the reduction of the resistances of the surface and the interface of the photoelectrode and enhanced the absorption spectrum in the UV–vis and near-infrared regions. The internal resistances and absorbance of the photoelectrode directly affected the power conversion efficiency. Green phosphor plays an important role towards the realization of high-efficiency dye-sensitized solar cells.

## Abbreviations

DSSC: dye-sensitized solar cells; FE-SEM: field emission-scanning electron microscopy; FF: fill factor; FTO: fluorine-doped SnO_2_ conducting glass plates; G2: (Mg Zn)Al_11_O_19_:Eu; G4: LaPO_4_:Ce Tb; IPCE: incident photon-to-current conversion efficiency; Jsc: short-circuit current density; Rct: charge-transfer resistance; Rs: bulk resistance; Voc: open-circuit voltage; η: energy conversion efficiency.

## Competing interests

The authors declare that they have no competing interests.

## Authors’ contributions

CKH and JJY performed UV–vis spectroscopic study and *I*-*V* result analysis. HSK fabricated the DSSCs. EMH performed the photoluminescence analysis. KHP drafted the manuscript. All authors read and approved the final manuscript.
